# Supplementary material for: Hybrid Models and Biological Model Reduction with PyDSTool
Source: PLoS Comput Biol. 2012 Aug 9;8(8):e1002628. doi: 10.1371/journal.pcbi.1002628 (PMC3415397; doi:10.1371/journal.pcbi.1002628)
Supplement: Text S4 — Complete source code for the PyDSTool package (version 0.88.120504). Includes API documentation and help files linking to web pages. This file is identical to the current public release on Sourceforge.net. (ZIP) [file pcbi.1002628.s004.zip › PyDSTool/html/PyDSTool.Toolbox.optimizers.criterion.facilities-module.html]

xml version="1.0" encoding="ascii"?


PyDSTool.Toolbox.optimizers.criterion.facilities


| Home | Trees | Indices | Help | PyDSTool |
| --- | --- | --- | --- | --- |

Package PyDSTool ::
Package Toolbox ::
Package optimizers ::
Package criterion ::
Module facilities

[hide private]

[frames] | no frames]

# Module facilities

source code

Proposes a way to create a composite criterion


|  |  |
| --- | --- |
| Functions | [hide private] |

|  |  |
| --- | --- |
| criterion(\*\*kwargs)  Creates a composite criterion based on the formal parameters : | source code |


|  |  |
| --- | --- |
| Function Details | [hide private] |

|  |  |
| --- | --- |
| criterion(\*\*kwargs) | source code |

Creates a composite criterion based on the formal parameters :

- iterations\_max indicates the maximum number of iteration
- ftol is the maximum relative change of the value function
- xtol is the maximum relative change of the parameters
- gtol is the maximum gradient

  


| Home | Trees | Indices | Help | PyDSTool |
| --- | --- | --- | --- | --- |

|  |  |
| --- | --- |
| Generated by Epydoc 3.0.1 on Fri May 4 15:24:05 2012 | http://epydoc.sourceforge.net |
